# Supplementary material for: Insulin-like Growth Factor-Binding Protein 2 in Severe Aortic Valve Stenosis and Pulmonary Hypertension: A Gender-Based Perspective
Source: Int J Mol Sci. 2024 Jul 27;25(15):8220. doi: 10.3390/ijms25158220 (PMC11312253; doi:10.3390/ijms25158220)
Supplement: Supplementary file 1 [file ijms-25-08220-s001.zip › ijms-3063453-supplementary.pdf]

1-year mortality<br>Male<br>Cox Regression Analysis	Univariate		Multivariate	
	**Hazard Ratio (95% CI)**	**p-value**	**Hazard Ratio (95% CI)**	**p-value**
Age	0.811 (0.529 — 1.241)	0.334		
Height	1.122 (0.448 — 2.814)	0.806		
Weight	1.164 (0.664 — 2.041)	0.596		
BMI	1.214 (0.668 — 2.204)	0.525		
NYHA ≥ III	1.672 (0.187 — 14.968)	0.646		
STS-Score	1.446 (0.839 — 2.491)	0.184		
Diabetes mellitus	1.160 (0.314 — 4.288)	0.824		
Arterial Hypertension	27.272 (0.046 — 16155.557)	0.310		
CVD	0.496 (0.160 — 1.538)	0.224		
CVD - 1 vessel	0.038 (0.000 — 31.533)	0.340		
CVD - 2 vessels	1.083 (0.237 — 4.944)	0.918		
CVD - 3 vessels	0.336 (0.043 — 2.604)	0.297		
Previous myocardial infarction	2.872 (0.360 — 22.373)	0.314		
Atrial fibrillation	0.603 (0.132 — 2.755)	0.514		
Pacemaker (before TAVR)	0.045 (0.000 — 1803.469)	0.567		
Malignancy	0.216 (0.028 — 1.675)	0.143		
Stroke (before TAVR)	2.150 (0.471 — 9.822)	0.323		
PAOD	0.044 (0.000 — 448.835)	0.507		
COPD	0.861 (0.111 — 6.669)	0.886		
LVEF	0.930 (0.569 — 1.520)	0.772		
LVEDD	0.085 (0.000 — 453486.200)	0.810		
IVSd	0.661 (0.367 — 1.189)	0.167		
AV Vmax	0.563 (0.139 — 2.288)	0.422		
AV dpmax	0.880 (0.381 — 2.029)	0.763		
AV dpmean	0.899 (0.372 — 2.168)	0.812		
TAPSE	1.176 (0.537 — 2.577)	0.685		
sPAP	2.228 (1.345 — 3.691)	0.002	1.717 (0.979 — 3.012)	0.059
AVI ≥ II°	0.037 (0.000 - 163.960)	0.441		
MVI ≥ II°	0.763 (0.206 — 2.819)	0.685		
TVI ≥ II°	0.932 (0.084 — 10.281)	0.954		
Creatinine	738.274 (4.079 — 13363.957)	0.013	4.362 (0.004 — 4686.232)	0.679
BNP	1.221 (0.800 — 1.864)	0.355		
HK	0.692 (0.420 — 1.143)	0.150		
HB	0.725 (0.443 — 1.186)	0.200		
CK	0.157 (0.013 — 1.860)	0.142		
IGF-BP2 (pre TAVR)	1.665 (1.185 — 2.340)	0.003	1.400 (1.037 — 1.891)	0.028
IGF-BP2 (24 hours post TAVR)	1.585 (1.136 — 2.211)	0.007	0.720 (0.350 — 1.481)	0.372
IGF-BP2 (96 hours post TAVR)	1.298 (0.935 — 1.801)	0.119		
IGF-BP2 (3 months post TAVR)	1.405 (0.836 — 2.363)	0.199		

Supplementary Table S1 Univariate and multivariate cox regression analysis detecting 1-year mortality in male sex

3-year mortality<br>Male<br>Cox Regression Analysis	Univariate		Multivariate	
	Hazard Ratio (95% CI)	p-value	Hazard Ratio (95% CI)	p-value
Age	0.879 (0.602 — 1.283)	0.505		
Height	1.179 (0.542 — 2.565)	0.678		
Weight	1.245 (0.782 — 1.981)	0.356		
BMI	1.287 (0.778 — 2.128)	0.326		
NYHA ≥ III	2.120 (0.248 - 18.158)	0.493		
STS-Score	1.172 (0.681 — 2.019)	0.567		
Diabetes mellitus	0.727 (0.209 — 2.530)	0.616		
Arterial Hypertension	3.599 (0.477 - 27.148)	0.214		
CVD	0.416 (0.160 — 1.080)	0.071	0.633 (0.227 — 1.764)	0.381
CVD - 1 vessel	0.312 (0.041 — 2.355)	0.259		
CVD - 2 vessels	0.704 (0.161 — 3.081)	0.642		
CVD - 3 vessels	0.471 (0.108 — 2.063)	0.318		
Previous myocardial infarction	2.116 (0.279 — 16.018)	0.468		
Atrial fibrillation	0.945 (0.308 — 2.900)	0.921		
Pacemaker (before TAVR)	0.045 (0.000 — 259.021)	0.483		
Malignancy	0.500 (0.144 - 1.742)	0.277		
Stroke (before TAVR)	1.510 (0.345 — 6.613)	0.584		
PAOD	0.721 (0.096 — 5.440)	0.751		
COPD	0.592 (0.078 — 4.465)	0.611		
LVEF	0.967 (0.637 — 1.468)	0.876		
LVEDD	2.916 (0.000 — 174907.801)	0.893		
IVSd	0.692 (0.403 — 1.190)	0.183		
AV Vmax	0.766 (0.238 — 2.469)	0.656		
AV dpmax	1.122 (0.581 — 2.168)	0.731		
AV dpmean	1.139 (0.556 — 2.334)	0.723		
TAPSE	1.217 (0.650 — 2.278)	0.539		
sPAP	1.835 (1.206 — 2.792)	0.005	1.450 (0.912 — 2.305)	0.117
AVI ≥ II°	0.511 (0.065 — 4.034)	0.524		
MVI ≥ II°	0.021 (0.000 — 75.777)	0.354		
TVI ≥ II°	0.481 (0.138 — 1.675)	0.250		
Creatinine	141.341 (0.558 — 35785.880)	0.080	1.142 (0.001 — 922.085)	0.969
BNP	1.056 (0.704 — 1.586)	0.791		
HK	0.703 (0.462 — 1.072)	0.102		
HB	0.783 (0.517 — 1.186)	0.249		
CK	0.520 (0.129 — 2.102)	0.359		
IGF-BP2 (pre TAVR)	1.593 (1.125 — 2.256)	0.009	1.531 (1.180 — 1.986)	0.001
IGF-BP2 (24 hours post TAVR)	1.529 (1.104 — 2.117)	0.011	0.812 (0.411 — 1.603)	0.548
IGF-BP2 (96 hours post TAVR)	1.299 (0.943 — 1.791)	0.110		
IGF-BP2 (3 months post TAVR)	1.247 (0.760 — 2.047)	0.382		

Supplementary Table S2 Univariate and multivariate cox regression analysis detecting 3-year mortality in male sex

5-year mortality<br>Male<br>Cox Regression Analysis	Univariate		Multivariate	
	Hazard Ratio (95% CI)	p-value	Hazard Ratio (95% CI)	p-value
Age	1.156 (0.808 — 1.653)	0.427		
Height	0.914 (0.482 — 1.734)	0.783		
Weight	1.166 (0.783 — 1.735)	0.451		
BMI	1.281 (0.836 — 1.961)	0.255		
NYHA ≥ III	1.276 (0.338 — 4.825)	0.719		
STS-Score	1.473 (0.991 — 2.190)	0.055	0.758 (0.327 — 1.758)	0.519
Diabetes mellitus	1.036 (0.414 — 2.596)	0.940		
Arterial Hypertension	2.704 (0.637 — 11.476)	0.177		
CVD	0.672 (0.302 — 1.498)	0.331		
CVD - 1 vessel	0.974 (0.334 — 2.842)	0.962		
CVD - 2 vessels	0.664 (0.198 — 2.220)	0.506		
CVD - 3 vessels	0.646 (0.222 — 1.883)	0.423		
Previous myocardial infarction	3.109 (0.729 — 13.259)	0.125		
Atrial fibrillation	1.255 (0.524 — 3.008)	0.610		
Pacemaker (before TAVR)	1.345 (0.317 — 5.713)	0.688		
Malignancy	0.941 (0.393 — 2.254)	0.891		
Stroke (before TAVR)	1.520 (0.455 - 5.084)	0.496		
PAOD	1.725 (0.515 — 5.774)	0.377		
COPD	0.773 (0.182 — 3.279)	0.727		
LVEF	0.991 (0.698 — 1.407)	0.961		
LVEDD	61.691 (0.000 — 134033.786)	0.511		
IVSd	0.697 (0.434 — 1.119)	0.135		
AV Vmax	0.967 (0.355 — 2.634)	0.947		
AV dpmax	1.266 (0.712 — 2.249)	0.422		
AV dpmean	1.266 (0.676 — 2.371)	0.462		
TAPSE	1.182 (0.691 — 2.020)	0.542		
sPAP	1.480 (1.041 — 2.105)	0.029	1.261 (0.795 — 2.000)	0.325
AVI ≥ II°	0.242 (0.032 — 1.824)	0.169		
MVI ≥ II°	0.913 (0.378 — 2.202)	0.839		
TVI ≥ II°	0.304 (0.071 — 1.294)	0.107		
Creatinine	491.644 (4.622 — 52298.685)	0.009	1.286 (0.000 - 570.455)	0.970
BNP	1.218 (0.433 — 3.431)	0.709		
HK	0.759 (0.536 — 1.075)	0.121		
HB	0.806 (0.572 — 1.136)	0.218		
CK	1.048 (0.686 — 1.600)	0.829		
IGF-BP2 (pre TAVR)	1.659 (1.201 — 2.291)	0.002	1.462 (1.075 — 1.988)	0.016
IGF-BP2 (24 hours post TAVR)	1.490 (1.092 —2.035)	0.012	0.858 (0.354 — 2.077)	0.734
IGF-BP2 (96 hours post TAVR)	1.340 (0.998 — 1.800)	0.052	1.125 (0.504 — 2.512)	0.774
IGF-BP2 (3 months post TAVR)	1.391 (0.964 — 2.008)	0.111		

Supplementary Table S3 Univariate and multivariate cox regression analysis detecting 5-year mortality in male sex

1-year mortality<br>Male + sPAP ≥ 40 mmHg<br>Cox Regression Analysis	Univariate		Multivariate	
	**Hazard Ratio (95% CI)**	**p-value**	**Hazard Ratio (95% CI)**	**p-value**
Age	0.686 (0.444 — 1.061)	0.090	0.622 (0.385 — 1.005)	0.052
Height	0.964 (0.293 — 3.173)	0.952		
Weight	1.296 (0.732 — 2.296)	0.374		
BMI	1.384 (0.782 — 2.450)	0.265		
NYHA ≥ III	0.663 (0.073 — 6.005)	0.715		
STS-Score	1.413 (0.640 — 3.117)	0.392		
Diabetes mellitus	2.802 (0.735 — 10.678)	0.131		
Arterial Hypertension	25.607 (0.017 — 390.596)	0.386		
CVD	0.529 (0.161 — 1.736)	0.294		
CVD - 1 vessel	0.044 (0.000 — 1082.083)	0.546		
CVD - 2 vessels	1.360 (0.293 — 6.308)	0.694		
CVD - 3 vessels	0.031 (0.000 — 10.328)	0.242		
Previous myocardial infarction	1.654 (0.211 — 12.984)	0.632		
Atrial fibrillation	0.360 (0.078 — 1.671)	0.192		
Pacemaker (before TAVR)	0.044 (0.000 — 1082.083)	0.546		
Malignancy	0.371 (0.047 — 2.901)	0.345		
Stroke (before TAVR)	2.481 (0.533 — 11.542)	0.247		
PAOD	0.042 (0.000 — 176.769)	0.456		
COPD	0.634 (0.081 — 4.955)	0.664		
LVEF	1.066 (0.632 — 1.795)	0.811		
LVEDD	0.004 (0.000 — 390.863)	0.691		
IVSd	1.145 (0.057 — 23.139)	0.930		
AV Vmax	0.440 (0.076 — 2.556)	0.360		
AV dpmax	0.835 (0.306 — 2.284)	0.726		
AV dpmean	0.740 (0.250 — 2.188)	0.586		
TAPSE	1.272 (0.619 — 2.616)	0.513		
AVI ≥ II°	0.041 (0.000 — 1234.707)	0.545		
MVI ≥ II°	0.658 (0.174 — 2.483)	0.537		
TVI ≥ II°	0.443 (0.096 — 2.053)	0.298		
Creatinine	112.859 (0.458 — 27780.164)	0.092	1.005 (0.001 —726.721)	0.999
BNP	1.050 (0.656 — 1.682)	0.839		
HK	0.822 (0.479 — 1.410)	0.476		
HB	0.890 (0.546 — 1.453)	0.642		
CK	0.093 (0.005 — 1.696)	0.109		
IGF-BP2 (pre TAVR)	1.356 (1.027 — 1.791)	0.032	1.458 (1.097 — 1.938)	0.009
IGF-BP2 (24 hours post TAVR)	1.402 (0.980 — 2.007)	0.065	1.186 (0.501 — 2.805)	0.698
IGF-BP2 (96 hours post TAVR)	1.215 (0.862 — 1.712)	0.266		
IGF-BP2 (3 months post TAVR)	1.235 (0.694 — 2.199)	0.473		

Supplementary Table S4 Univariate and multivariate cox regression analysis detecting 1-year mortality in male sex with an sPAP ≥ 40 mmHg

3-year mortality<br>Male + sPAP ≥ 40 mmHg<br>Cox Regression Analysis	Univariate		Multivariate	
	**Hazard Ratio (95% CI)**	**p-value**	**Hazard Ratio (95% CI)**	**p-value**
Age	0.744 (0.491 — 1.127)	0.163		
Height	1.184 (0.420 — 3.337)	0.749		
Weight	1.391 (0.847 — 2.284)	0.193		
BMI	1.458 (0.877 — 2.424)	0.146		
NYHA ≥ III	0.663 (0.073 — 6.005)	0.715		
STS-Score	1.018 (0.476 — 2.178)	0.963		
Diabetes mellitus	1.963 (0.550 — 7.008)	0.299		
Arterial Hypertension	2.431 (0.319 — 18.512)	0.391		
CVD	0.518 (0.187 — 1.432)	0.205		
CVD - 1 vessel	0.876 (0.115 — 6.676)	0.898		
CVD - 2 vessels	0.928 (0.209 — 4.118)	0.922		
CVD - 3 vessels	0.185 (0.024 — 1.412)	0.104		
Previous myocardial infarction	1.230 (0.161 — 9.380)	0.842		
Atrial fibrillation	0.390 (0.110 — 1.385)	0.145		
Pacemaker (before TAVR)	0.044 (0.000 — 173.791)	0.459		
Malignancy	0.565 (0.127 — 2.506)	0.452		
Stroke (before TAVR)	1.947 (0.436 — 8.695)	0.383		
PAOD	0.569 (0.075 — 4.331)	0.586		
COPD	0.442 (0.058 — 3.363)	0.430		
LVEF	1.163 (0.729 — 1.855)	0.527		
LVEDD	0.000 (0.000 — 215340.439)	0.402		
IVSd	0.759 (0.437 — 1.319)	0.329		
AV Vmax	0.356 (0.073 — 1.742)	0.202		
AV dpmax	0.912 (0.396 — 2.097)	0.828		
AV dpmean	0.848 (0.340 — 2.114)	0.724		
TAPSE	1.343 (0.749 — 2.410)	0.322		
AVI ≥ II°	0.997 (0.124 — 8.004)	0.998		
MVI ≥ II°	0.424 (0.120 — 1.504)	0.184		
TVI ≥ II°	1.047 (0.147 — 7.434)	0.964		
Creatinine	25.679 (0.059 — 11205.826)	0.295		
BNP	0.868 (0.537 — 1.405)	0.565		
HK	0.785 (0.492 — 1.253)	0.310		
HB	0.937 (0.616 — 1.426)	0.762		
CK	0.533 (0.135 — 2.098)	0.368		
IGF-BP2 (pre TAVR)	1.317 (0.991 — 1.750)	0.058	1.387 (1.053 — 1.827)	0.020
IGF-BP2 (24 hours post TAVR)	1.348 (0.945 — 1.922)	0.099	0.835 (0.381 — 1.833)	0.653
IGF-BP2 (96 hours post TAVR)	1.227 (0.880 — 1.712)	0.228		
IGF-BP2 (3 months post TAVR)	1.077 (0.616 — 1.882)	0.795		

Supplementary Table S5 Univariate and multivariate cox regression analysis detecting 3-year mortality in male sex with an sPAP ≥ 40 mmHg

5-year mortality<br>Male + sPAP ≥ 40 mmHg<br>Cox Regression Analysis	Univariate		Multivariate	
	Hazard Ratio (95% CI)	p-value	Hazard Ratio (95% CI)	p-value
Age	0.883 (0.587 — 1.330)	0.552		
Height	0.965 (0.402 — 2.314)	0.936		
Weight	1.380 (0.865 — 2.202)	0.177		
BMI	1.513 (0.943 — 2.429)	0.086	1.702 (0.966 — 2.999)	0.066
NYHA ≥ III	0.500 (0.100 — 2.496)	0.398		
STS-Score	1.056 (0.546 — 2.046)	0.871		
Diabetes mellitus	2.133 (0.703 — 6.470)	0.181		
Arterial Hypertension	1.445 (0.333 — 6.265)	0.623		
CVD	0.626 (0.254 — 1.546)	0.310		
CVD - 1 vessel	0.712 (0.095 — 5.344)	0.741		
CVD - 2 vessels	1.087 (0.316 — 3.736)	0.895		
CVD - 3 vessels	0.460 (0.133 — 1.586)	0.219		
Previous myocardial infarction	2.128 (0.488 — 9.284)	0.315		
Atrial fibrillation	0.721 (0.273 — 1.900)	0.508		
Pacemaker (before TAVR)	0.601 (0.080 — 4.519)	0.621		
Malignancy	0.677 (0.197 — 2.328)	0.536		
Stroke (before TAVR)	2.474 (0.716 — 8.548)	0.152		
PAOD	0.917 (0.212 — 3.976)	0.908		
COPD	0.675 (0.156 — 2.928)	0.600		
LVEF	1.153 (0.767 — 1.734)	0.492		
LVEDD	0.006 (0.000 — 3647.865)	0.572		
IVSd	0.761 (0.456 — 1.272)	0.298		
AV Vmax	0.359 (0.078 — 1.642)	0.187		
AV dpmax	0.813 (0.363 — 1.822)	0.616		
AV dpmean	0.783 (0.328 — 1.869)	0.582		
TAPSE	1.262 (0.760 — 2.096)	0.369		
AVI ≥ II°	0.696 (0.090 — 5.370)	0.728		
MVI ≥ II°	0.561 (0.201 — 1.561)	0.268		
TVI ≥ II°	0.189 (0.043 — 0.823)	0.027	0.201 (0.043 — 0.926)	0.040
Creatinine	16.117 (0.038 — 6780.809)	0.367		
BNP	0.927 (0.628 — 1.369)	0.703		
HK	0.793 (0.523 — 1.202)	0.274		
HB	0.922 (0.635 — 1.339)	0.668		
CK	1.010 (0.684 — 1.492)	0.960		
IGF-BP2	1.346 (1.024 — 1.768)	0.033	1.649 (1.162 — 2.339)	0.005
IGF-BP2 (24 hours post TAVR)	1.332 (0.942 — 1.883)	0.105		
IGF-BP2 (96 hours post TAVR)	1.274 (0.936 — 1.733)	0.124		
IGF-BP2 (3 months post TAVR)	1.335 (0.873 — 2.042)	0.182		

Supplementary Table S6 Univariate and multivariate cox regression analysis detecting 5-year mortality in male sex with an sPAP ≥ 40 mmHg
